# OCT3/4 expression is correlated with the invasion of gastric carcinoma

**DOI:** 10.3892/ol.2014.2112

**Published:** 2014-05-07

**Authors:** NA LI, WEIQIANG WANG, BIN XU, HONGYUN GONG

**Affiliations:** 1 Department of Oncology, Renmin Hospital of Wuhan University, Wuhan, Hubei 430060, P.R. China; 2 Department of Gastroenterology, 281st Hospital of the PLA, Qinhuangdao, Hebei 066100, P.R. China

**Keywords:** OCT3/4, gastric carcinoma, RNA interference, cell invasion

## Abstract

The present study aimed to evaluate the effect of OCT3/4 on the invasion and metastasis ability of gastric cancer. First, the expression level of OCT3/4 was detected in gastric cancer tissues of different tumor-node-metastasis stages. Furthermore, the correlation between the expression of OCT3/4 and the invasion ability of gastric cancer cells, and the probable regulatory mechanism were observed by RNA interference of OCT3/4 in gastric cancer cell strain MKN28, so as to provide the molecular mechanism for the occurrence and development of gastric cancer. The present study found the expression of OCT3/4 in gastric carcinoma tissues (22.56±8.72%) was markedly higher compared with that in para-cancer tissue (1.12±0.18%) (P<0.01). The expression of OCT3/4 was associated with the infiltration degree, and demonstrated an increasing tendency from Tis-T4 stages or from N0-N3. The expression of OCT3/4 in M0 tissues was markedly lower than that in M1 tissues (P<0.01). The level of OCT3/4 was markedly decreased following transfection with OCT3/4 small interfering (si)RNA (P<0.01). The number of cell clones was reduced in a dose-dependent manner following transfection with increasing levels of siRNA, and the number of cells that permeated through the filter membrane was also decreased. It may be concluded that the expression of OCT3/4 increases along with the degree of the infiltration and metastasis of gastric carcinoma, and that OCT3/4 siRNA inhibits the invasion of gastric carcinoma cells.

## Introduction

Studies within recent years have indicated that tumor stem cells are significant in tumor formation and progression. Tumor stem cells comprise some of the tumor tissue found to possess characteristics associated with stem cells, i.e., the potential to self-renew and differentiate into various cell types (1–3). Moreover, they are the source of the growth, metastasis and relapse of tumors. At present, tumor stem cells have been confirmed in gastric cancer cells, and they are relevant to the formation, development and prognosis of gastric cancer (4,5). Stem cell transcription factor Oct-3/4 is indispensable for the maintenance of cell totipotency, which is involved in regulating growth of the embryo and tissues and self-renewal of embryonic stem cells or primordial germ cells, whereas in differentiated or mature tissues, its expression level is reduced or even completely absent (6,7). It has been reported that the expression of OCT3/4 in gastric cancer tissues is associated with the invasion ability and prognosis of gastric cancer (8). In order to evaluate the effect of OCT3/4 on the invasion and metastasis ability of gastric cancer, the present study first detected the expression level of OCT3/4 in the gastric cancer tissues of different tumor-node-metastasis (TNM) stages. Furthermore, the correlation between the expression of OCT3/4 and the invasion ability of gastric cancer cells, and the probable regulatory mechanism were observed by RNA interference of OCT3/4 in gastric cancer cell strain MKN28, so as to provide the molecular mechanism for the occurrence and development of gastric cancer.

## Materials and methods

### Specimen source

The study involved 126 gastric cancer specimens surgically obtained between 2003 and 2009 from 73 males and 53 females, with an average age of 61.3±11.7 years (range, 48–72 years). All surgical specimens were presented as paraffin sections, and adjacent non-cancerous tissues were used as controls. The study was approved by the ethics committee of Wuhan University (Wuhan, China) and patients provided written informed consent.

For gastric cancer staging, the 7th International Union Against Cancer TNM classification was adopted (9). T represented tumor invasion depth, with Tis (primary tumor only confined to mucous layer) found in 14 cases, T1 (invasion to or below mucous layer) in 23 cases, T2 (invasion to muscular layer or plasma layer) in 35 cases, T3 (invasion through plasma layer) in 38 cases and T4 (invasion to adjacent structures or intracavitary spreading to esophagus and duodenum) in 16 cases. N represented the extent of lymph node metastasis, with N0 (no pathological findings of involvement in the dissected lymph nodes ≥15 in number) found in 35 cases, N1 (1–6 regional lymph node metastases) in 48 cases, N2 (7–15 regional lymph node metastases) in 28 cases and N3 (≥16 regional lymph node metastases) in 15 cases. M represented distant metastasis, with M0 (no distant metastasis) found in 98 cases and M1 (distant metastasis to the pancreatic gland, mesentery or abdominal paraaortic lymph nodes) in 28 cases.

### Cell strains and antibodies

The human gastric cancer MKN28 cell strain was maintained in liquid nitrogen at the Cancer Research Laboratory of Wuhan University (Wuhan, China). Standard RPMI-1640 medium containing 10% fetal bovine serum was purchased from Gibco-BRL (San Francisco, CA, USA). Rabbit anti-human OCT3/4 (38 kDa) multiclone antibodies were purchased from Santa Cruz Biotechnology, Inc. (Santa Cruz, CA, USA), while rabbit anti-goat secondary antibodies coated with horseradish peroxidase were purchased from Wuhan Boster Biological Technology, Ltd. (Wuhan, China). Kits for the immunohistochemical staining of labeled streptavidin biotin (LSAB; Dako, Glostrup, Denmark), liposome Oligofectamine (Invitrogen Life Technologies, Carlsbad, CA, USA) and OCT3/4 small interfering (si)RNA double-stranded oligonucleotide, and Transwell chamber models (Chemicon, Temecula, CA, USA) were used. Western blotting kits were purchased from Wuhan Boster Biological Technology, Ltd.

### Construction of OCT3/4 an siRNA sequence and transfection of the gastric cancer MKN28 strain

Three OCT3/4 siRNA oligonucleotides obtained from Thermo Fisher Scientific (Waltham, MA, USA) were matched with a human OCT3/4 cDNA sequence from GenBank following sequence identification and contrasting with BLAST (http://www.ncbi.nlm.nih.gov/nucleotide/553727228?report=genbank&log$=nuclalign&blast_rank=16&RID=KJXJYKX501R). The siRNA sense sequence was AAGGAUGUGGUCCGAGUGUGG. By contrast, the siRNA negative control sense was formulated and synthesized as AAGAACGGCAUCAAGGUGAAC. OCT3/4 siRNA was transfected into MKN28 cells (1×10^5^ cells/ml) at concentrations of 6.25–100 nM using Oligofectamine. The group design consisted of a blank control group (Con-B group), an empty vector group (Con-A group), transfection groups (groups S1-S3) and a negative control group (Sn group). With the exception of the use of isocyatic phosphate-buffered saline and an empty vector for the Con-B and Con-A groups, respectively, the subsequent treatment of each group was the same.

### Immunohistochemical detection of OCT3/4 expression in gastric cancer tissues

LSAB kits were used for immunohistochemical staining, according to the manufacturer’s instructions. Briefly, OCT3/4 primary antibody (1:200 dilution) and biotin-labeled secondary antibody (1:10,000 dilution) was added. The slices were visualized for 5 minutes following staining, then restained with hematoxylin and sealed. Positive cells were stained brown in the nucleolus or cytolymph. Imag-pro-plus software was employed to measure the percentage of the positively-stained cell area compared with the reference strain area.

### Western blot analysis

MKN28 cells in the exponential growth phase were lysed by adding radioimmunoprecipitation assay buffer (Wuhan Boster Biological Technology, Ltd.). Following centrifugation at 30,000 × g at 4°C for 5 min, the supernatant was obtained to determine the protein level by the bicinchoninic acid method. Successively, 50 μg protein was extracted and mixed with 2× loading buffer prior (Wuhan Boster Biological Technology, Ltd.) to denaturation at 100°C for 5 min. Following separation by SDS-PAGE (Wuhan Boster Biological Technology, Ltd.), the proteins were transferred to a nitrocellulose filter (Wuhan Boster Biological Technology, Ltd.), where they bound to specific antibodies and relative secondary antibodies, were visualized by staining with enhanced chemiluminescence (Wuhan Boster Biological Technology, Ltd.) and were exposed, developed and fixed on X-ray films (Wuhan Boster Biological Technology, Ltd.). Gray-scale analysis was performed using BandScan software (ProZyme, Inc., Hayward, CA, USA).

### Soft agar colony formation assay

The MKN28 cells at the exponential phase were suspended in a given concentration of 1×10^3^ cells/ml. A mixture of 5% soft agar (Hyclone, Logan, MA, USA) plus medium at a ratio of 1:9 was added to the culture dishes and kept at room temperature for solidification. Additionally, 1.5 ml cell suspension was mixed with an equivalent volume of 5% soft agar in a 5% CO_2_ incubator at 37°C for 2 weeks. Colony formation conditions and rates were observed using the following formula: Colony formation rate (%) = (colony number / incubated cell number) × 100.

### In vitro cellular invasion test

Transwell chamber models were employed to perform an *in vitro* cell invasion test. Cell suspension with a given concentration of 1×10^5^ cells/ml was prepared, 50 μl of which was added to the upper chamber. At 24 h post-incubation, the cells on the upper chamber were wiped off and the number that had migrated through the permeable membrane were counted by formalin (10%) fixation and Giemsa dyeing.

### Statistical analysis

The data are expressed as the mean ± SD. The χ^2^ test and two-tailed t-test were conducted with SPSS version 16.0 (SPSS, Inc., Chicago, IL, USA). P<0.05 was considered to indicate a statistically significant difference.

## Results

### Correlation between OCT3/4 expression and the invasion and metastasis of gastric carcinoma

**Figure 1:**
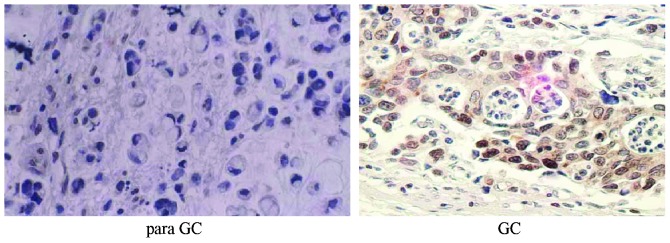
Immunohistochemical detection of OCT3/4 expression in gastric cancer tissues and adjacent non-cancerous tissues (LSAB; magnification, ×100). OCT3/4 exhibited extremely low levels of expression in the adjacent non-cancerous tissues, but was present in higher levels as brown staining in the cancer cell nuclei. LSAB, labeled streptavidin biotin.

The immunohistochemical results showed an extremely low level of OCT3/4 in the adjacent non-cancerous tissues (1.12±0.18%) compared with that in the cancer tissues (22.56±8.72%) of the 126 gastric cancer patients (P<0.01). Positive reactivity of OCT3/4 was mainly restricted to the cell nuclei (Fig. 1). The expression of OCT3/4 was associated with the invasion depth of the gastric cancer, which presented a gradual rising trend from Tis-T4 stages. OCT3/4 had significantly higher expression in the T2, T3 and T4 stages compared with the Tis stage, with >10-fold higher expression in the T4 stage compared with the Tis stage. The expression of OCT3/4 was observed to gradually increase as the extent of the lymph node metastasis increased. A significant difference was also observed between N2 and N0 (P<0.05). Likewise, ~5-fold higher expression was found in N3 category compared with the N0 category. With regard to distant metastasis, higher OCT3/4 expression was observed in M1 compared with M0 (P<0.01) (Table I).

### Effect of siRNA on OCT3/4 expression

**Figure 2:**
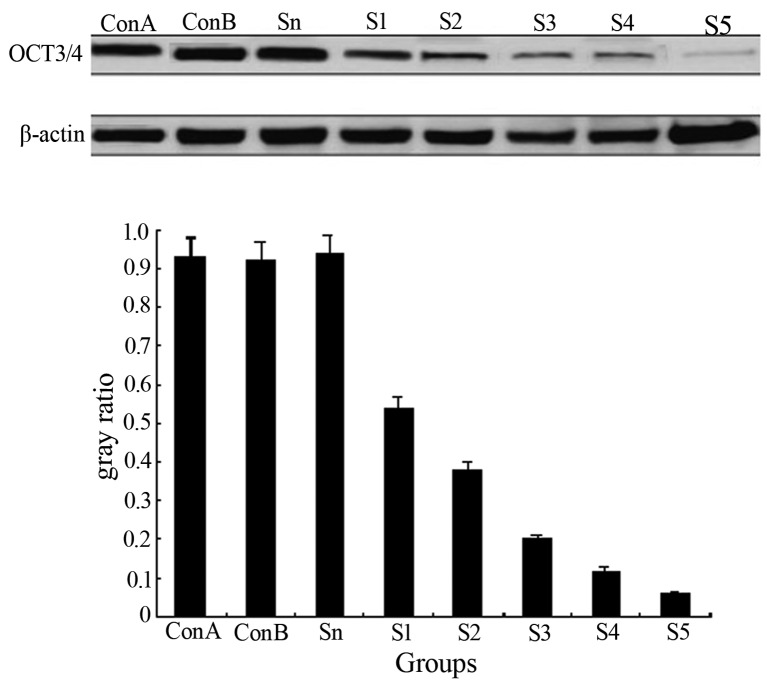
Correlation between OCT3/4 expression and the extent of gastric cancer invasion. small interfering (si)RNA suppressed OCT3/4 expression in a concentration-dependent manner: S1, 6.25 nM; S2, 12.5 nM; S3, 25 nM; S4, 50 nM; and S5, 100 nM.

Western blot analysis showed high OCT3/4 expression in the Con-B, Sn and Con-A groups, but the difference among the three groups was insignificant (P>0.05). OCT3/4 expression in each transfection group was downregulated significantly (P<0.01), particularly in the S3 group where the OCT3/4 level had decreased by 90.6%, compared with the control group (Fig. 2). The results indicated that siRNA transfection in the MKN28 cells was able to suppress OCT3/4 expression.

### Effect of OCT3/4 siRNA on the anchorage-independent growth of MKN28 cells

**Figure 3:**
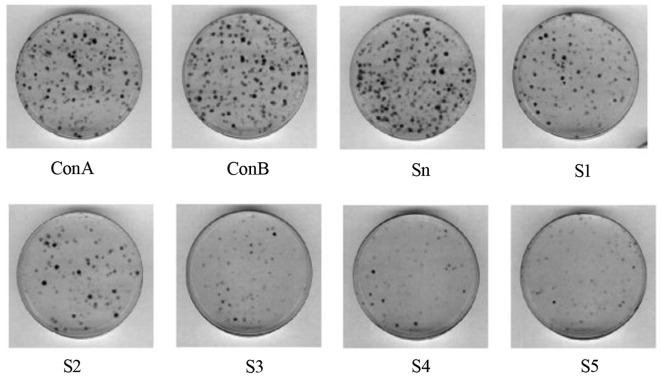
The colony formation rate was observed to gradually decrease following transfection of the MKN28 cells with various concentrations of siRNA3 (0, 6.25, 12.5, 25, 50 and 100 nM). Con A, empty vector; Con B, blank control; S1, 6.25 nM; S2, 2.5 nM; S3, 25 nM; S4, 50 nM; and S5, 100 nM.

As S3 was indicated to be the most effective interference sequence targeted to OCT3/4, OCT3/4 siRNA3 was selected to continue the study. The soft agar colony formation assay revealed that the MKN28 cells could form colonies spontaneously in an *in vitro* culture system. Following transfection of the MKN28 cells with different concentrations of siRNA3 (0, 6.25, 12.5, 25, 50, and 100 nM), the colony formation rate was observed to gradually decrease in a dose-dependent manner as the transfection volume increased (Fig. 3).

### Effect of OCT3/4 interference on the invasion capacity of MKN28 cells

**Figure 4:**
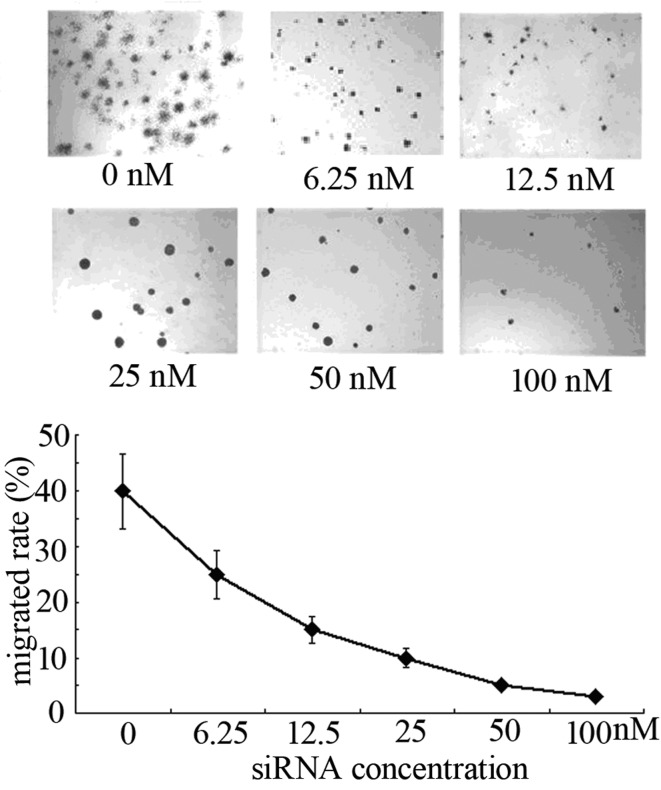
The migration rate of MKN28 cells decreased following OCT3/4 interference, which was associated with the siRNA concentration.

At 48 h post-transfection of MKN28 cells with different concentrations of siRNA3, Transwell chamber models were employed to detect their invasion ability. The number of cells that had migrated through the filter membrane was shown to have decreased significantly following OCT3/4 interference and was associated with the siRNA concentration (P<0.01; Fig. 4).

## Discussion

With deeper study into tumor stem cells in recent years, the possible existence of gastric cancer stem cells has come to light. OCT3/4 is a major molecular marker for stem cells (10). OCT3/4 is a POU transcription factor that is encoded by the POU5F1 gene located on human chromosome 6q21.3 and contains 324 amino acids at a molecular weight of 38 kDa (11). By binding to target gene promoter or octamer sequence ATGCAAAT in the enhancer region, OCT3/4 is activated to start transcription. OCT3/4 is a transcription factor required to maintain the self-renewal of embryonic stem cells and primordial germ cells (12); in well-differentiated tissues its expression is reduced or even completely absent (7,13). Certain studies have indicated that OCT3/4 is highly expressed in multiple tumor tissues or tumor cells, such as germinal cell tumors of the testis (14) and renal medullary (15), esophageal (16) and breast (17) carcinoma, and that this expression is correlated with the diagnosis and prognosis of the tumor.

A recent study showed that the expression of OCT3/4 in gastric cancer tissues was correlated with the prognosis of the gastric cancer patients (8). In the present study, OCT3/4 was mainly located in the cell nuclei, with a far higher level in the cancer tissues than in the adjacent non-cancerous tissues. The expression of OCT3/4 followed a rising trend with the worsening of tumor invasion, lymph node metastasis and distant metastasis (Fig. 1; Table I). Therefore, it can be initially concluded that OCT3/4 plays a certain role in the invasion and metastasis of gastric cancer and is significant for the instruction of the TNM staging of gastric cancer. Chen *et al* (18) used quantitative PCR to detect the mRNA expression of OCT3/4 in 62 cases of gastric cancer. The study reported higher OCT3/4 expression in the gastric cancer tissues compared with the adjacent non-cancerous tissues, atrophic gastritis or gastric ulcer samples. The study concluded that the expression of OCT3/4 was correlated with the extent of gastric cancer differentiation, but not with patient age, gender, tumor size, TNM staging or lymph node metastasis. In contrast, the present study highlighted the associations between the expression level of OCT3/4 in gastric cancer tissues with the different degrees of invasion, lymph node metastasis and distant metastasis. Significant differences in OCT3/4 level were found in the gastric cancer tissues with different extents of invasion, lymph node metastasis and distant metastasis (P<0.01).

To further examine the role of OCT3/4 in the invasion and metastasis of gastric cancer, the present study used specific RNA interference to silence OCT3/4, and employed a soft agar colony formation assay and Transwell chamber models to detect cell anchorage-independence and invasion capability. Cell anchorage dependence refers to the fact that normal cells must attach to a solid surface for growth, while tumor cells have the properties of anchorage-independent growth. Soft agar colony formation assays are able to determine the anchorage-independence and the degree of malignancy of tumor cells (19). A stronger invasion ability in cancer cells gives rise to more colony formation. The present study indicated that OCT3/4 siRNA could suppress the colony formation of MKN28 cells on soft agar in a concentration-dependent manner (Fig. 3). Accordingly, interfering with OCT3/4 can suppress the invasion of MKN28 cells. The migration and invasion ability of tumor cells is associated with the tumor microenvironment and the extracellular matrix, thus a Transwell chamber model simulated in accordance with the extracellular matrix is a reliable method for studying cell invasion ability (20). The present study found that subsequent to the interference of OCT3/4, the number of cells that migrated through Transwell membrane was significantly decreased in a siRNA concentration-dependent manner (Fig. 4).

The present study found that OCT3/4 in gastric cancer may play a role similar to that of oncogenes. The expression level of OCT3/4 may predict the malignant potential of gastric cancer, and downregulation of its expression may suppress the cell invasion ability. Asadi *et al* (21) found that a novel spliced variant of OCT4 designated as OCT4B1 was highly expressed in a gastric cancer cell strain, and that downregulation of its expression accelerated cell apoptosis. The study also described OCT4B1 as a novel tumor marker with potential value in the diagnosis and treatment of gastric cancer.

In conclusion, the present study showed that the expression of OCT3/4 increased with the worsening of gastric cancer invasion and metastasis, and interfering with OCT3/4 therefore attenuated the invasion ability of the gastric cancer cell MKN28 strain. These findings indicate that OCT3/4 plays a vital role in regulating the invasion and metastasis of gastric cancer. Detection of OCT3/4 is conducive to judging the malignant potential and prognosis of gastric cancer. Furthermore, the downregulation of OCT3/4 reduces the invasion and metastasis of gastric cancer, therefore, OCT3/4 is expected to be a molecular target for the treatment of this disease. The present study is specific to surface phenomenon observations and sets the stage for a deeper investigation into the role of OCT3/4 in the occurrence, development and treatment of gastric cancer and the possible mechanism behind this.

## Figures and Tables

**Table I:** Expression of OCT3/4 in gastric cancer with different invasion degree.

	n	OCT3/4, %^a^	F-value	P-value
Depth of infiltration				
Tis	14	3.14±0.47		
T1	23	9.21±1.31	T1/Tis, 6.069	<0.01
T2	35	16.23±2.03	T2/T1, 7.013	<0.01
T3	36	26.01±3.46	T3/T2, 9.7878	<0.01
T4	18	44.46±5.30	T4/T3, 18.449	<0.01
Lymph node metastasis				
N0	35	5.46±2.39		
N1	48	6.63±1.67	N1/N0, 1.169	0.039
N2	28	13.69±2.80	N2/N1, 7.057	<0.01
N3	15	26.86±4.10	N3/N2, 13.167	<0.01
Distant metastasis				
M0	98	11.12±9.39	M1/M0, 149.242	<0.01
M1	28	36.35±10.47		

a Data are presented as the mean ± standard deviation.
